# Supplementary figures and images for: Efficient transposon mutagenesis mediated by an IPTG-controlled conditional suicide plasmid
Source: BMC Microbiol. 2018 Oct 24;18:158. doi: 10.1186/s12866-018-1319-0 (PMC6201506; doi:10.1186/s12866-018-1319-0)

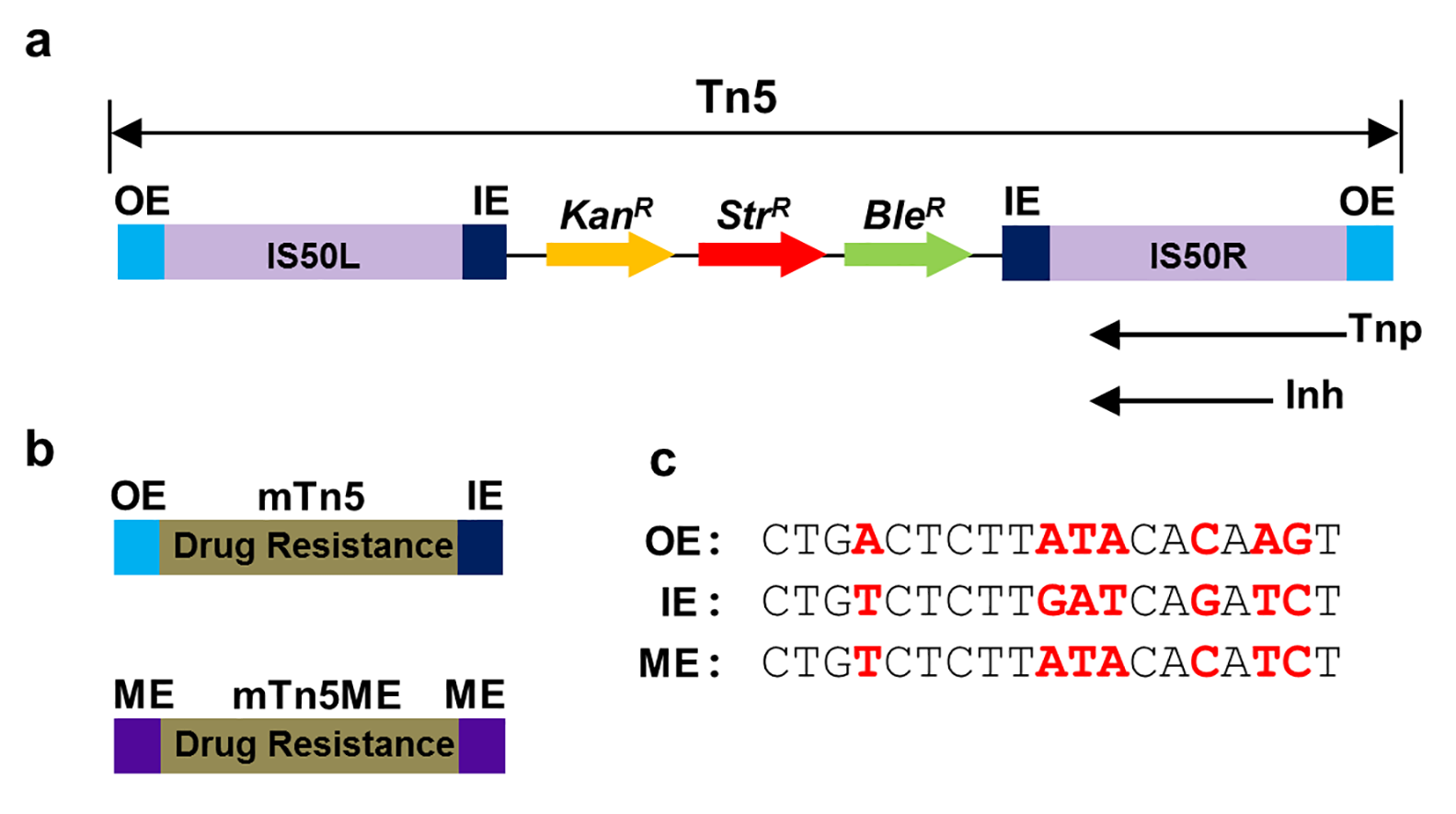

Supplement: Supplementary file 1 — Figure S1. Tn5 transposons. (a) Full-length Tn5. The full-length Tn5 contains two inverted IS50 elements at its ends. Only one of them encodes an active Tnp and an Inh (Inhibitor of Tnp). KanR, kanamycin-resistance gene; StrR, streptomycin-resistance gene; and BleR, bleomycin-resistance gene. (b) mTn5s. Top, an mTn5 with an OE and an IE at the termini. Bottom, an mTn5 with MEs at the ends. (c) Comparison of OE, IE and ME, with their polymorphisms highlighted in red. (TIF 4125 kb) [file 12866_2018_1319_MOESM1_ESM.tif]

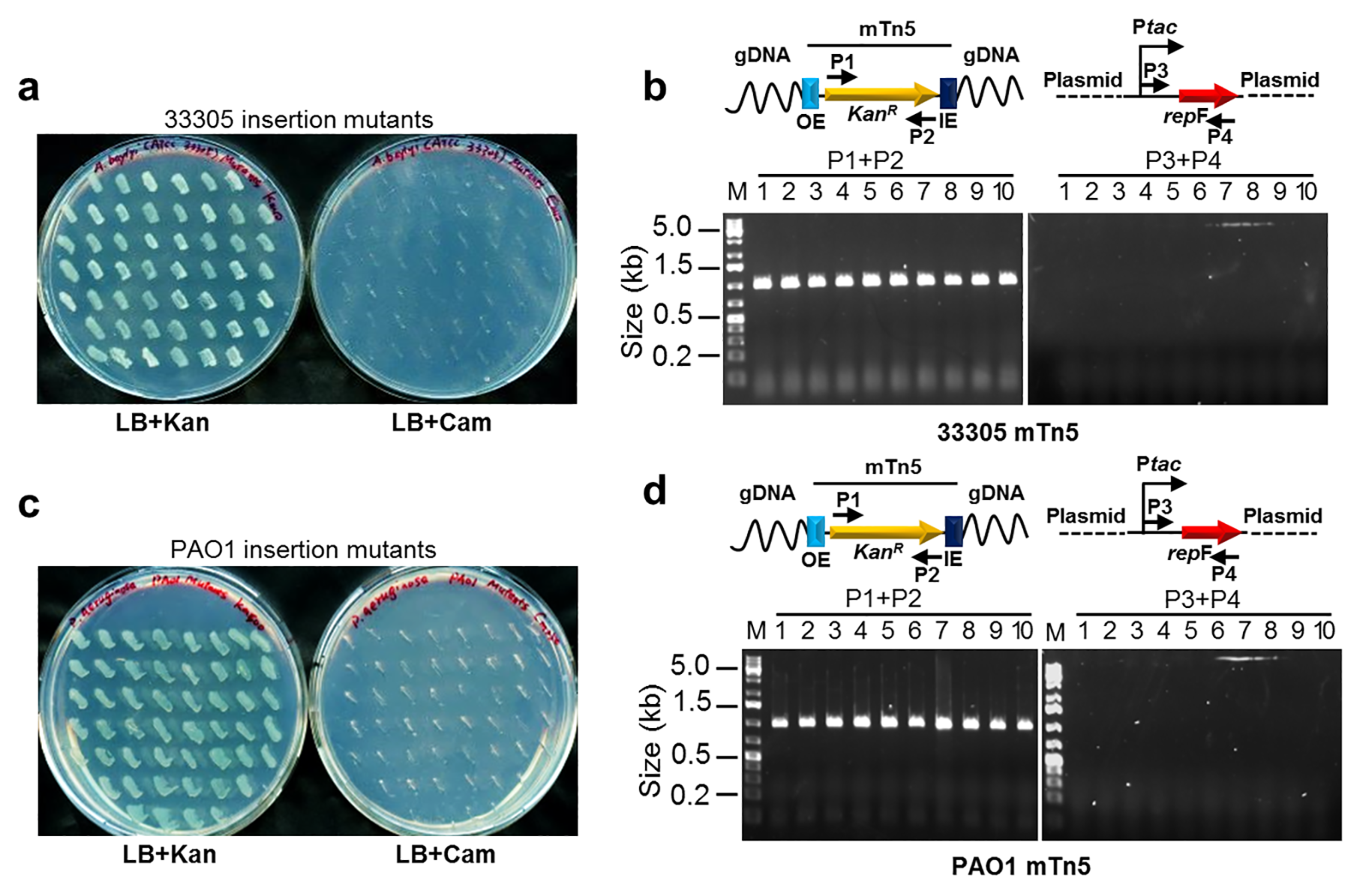

Supplement: Supplementary file 2 — Figure S2. Confirmation of mTn5 transposition events in A. baylyi and in P. aeruginosa. (a) Colony restreaking assay of A. baylyi. 100 SucRKanR colonies of A. baylyi were restreaked on LB + Kan and LB + Cam plates, and all were found to be KanRCamS. 50 are shown here. (b) Colony PCR of 10 restreaked A. baylyi clones with the indicated primers. All were mTn5-positive and plasmid-negative. (c) Colony restreaking assay of P. aeruginosa. 100 SucRKanR colonies of P. aeruginosa were restreaked on LB + Kan and LB + Cam plates, and all were found to be KanRCamS. 50 are shown here. (d) Colony PCR of ten restreaked P. aeruginosa clones with primers indicated in the diagram. All were mTn5-positive and plasmid-negative. (TIF 6384 kb) [file 12866_2018_1319_MOESM2_ESM.tif]

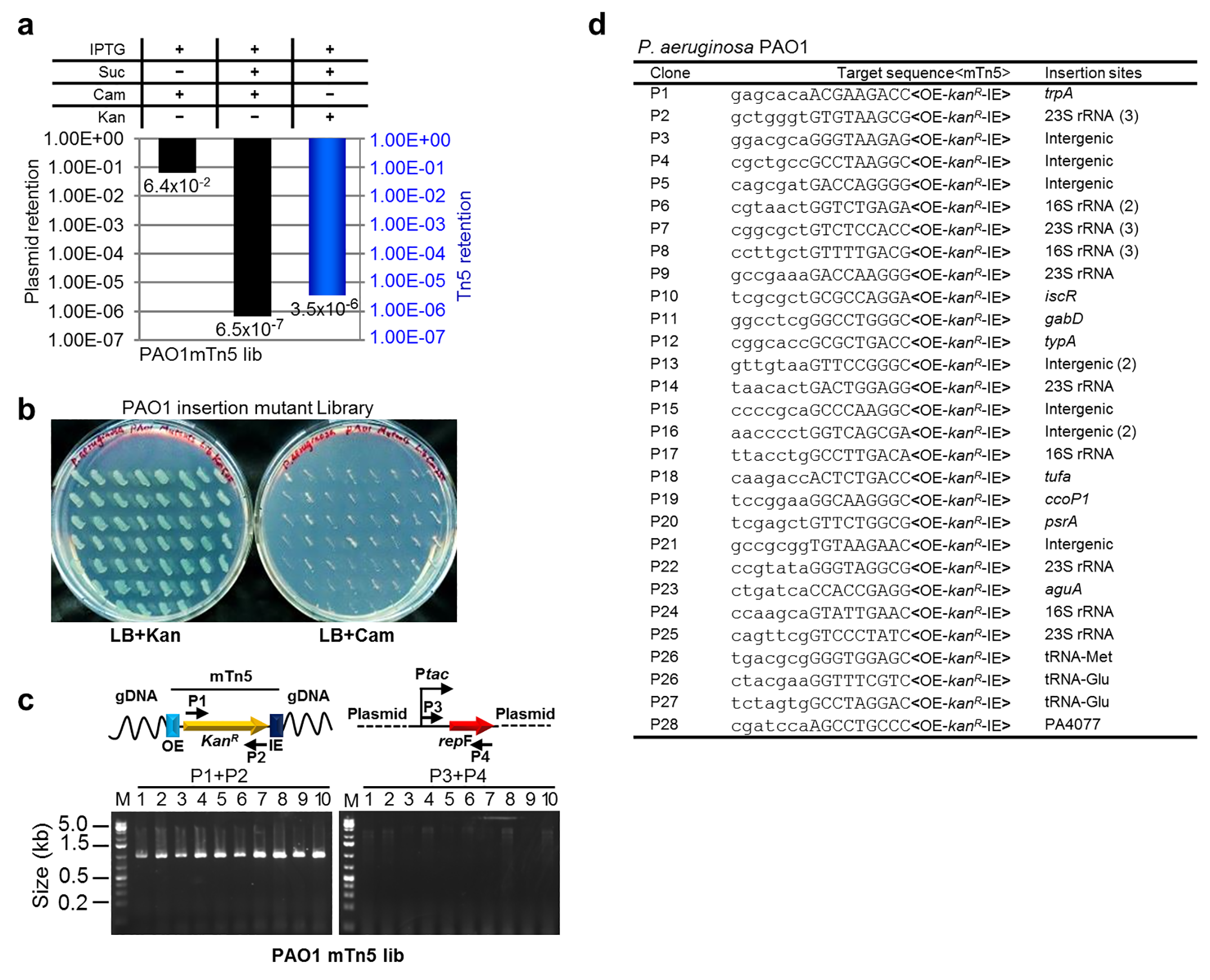

Supplement: Supplementary file 3 — Figure S3. A transposon insertion library of P. aeruginosa PAO1 generated with pSNC-mTn5. (a) Plasmid and transposon retention frequencies of the mTn5 insertion library of P. aeruginosa PAO1. (b) Colony restreaking assay. 100 random SucRKanR colonies were restreaked on LB + Kan and LB + Cam plates. 100/100 were found to be KanRCamS and 50 are shown here. (c) Colony PCR of ten restreaked clones in (b). All were found to be mTn5-positive and plasmid-negative. (d) mTn5 insertion sites of 37 mutant clones from the transposon insertion library of P. aeruginosa. Identical clones are only shown once, and their numbers are indicated in parenthesis. (TIF 1363 kb) [file 12866_2018_1319_MOESM3_ESM.tif]

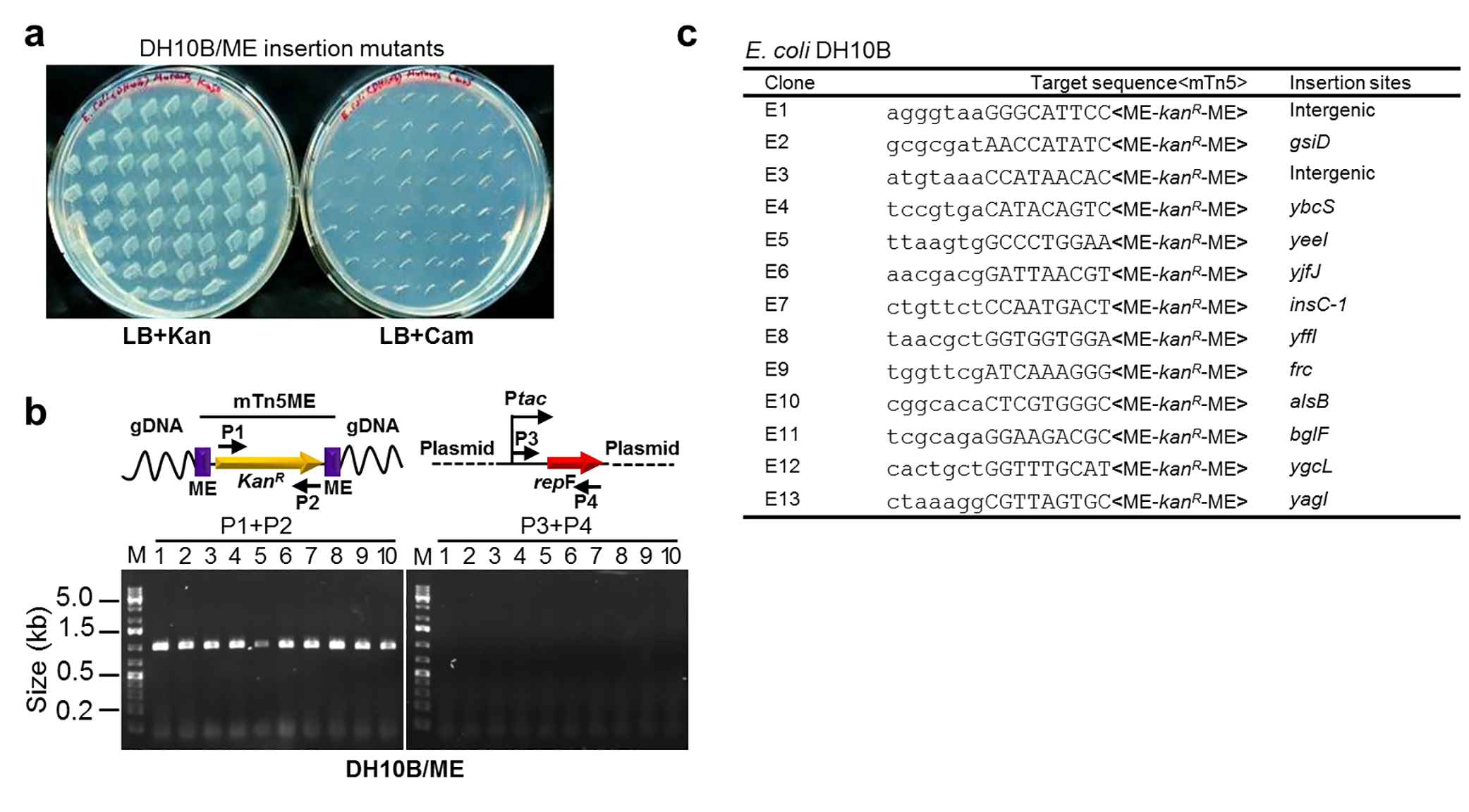

Supplement: Supplementary file 4 — Figure S4. Confirmation of mTn5ME transposition events in E. coli DH10B. (a) Colony restreaking. 100 random SucRKanR colonies of E. coli were restreaked on LB + Kan and LB + Cam plates. 100/100 were found to be KanRCamS and 50 restreaked colonies are shown here. (b) Colony PCR of ten restreaked clones in (a). All were found to be Tn5-positive and plasmid-negative. (c) Tn5 insertion sites of 13 independent DH10B clones. (TIF 7051 kb) [file 12866_2018_1319_MOESM4_ESM.tif]

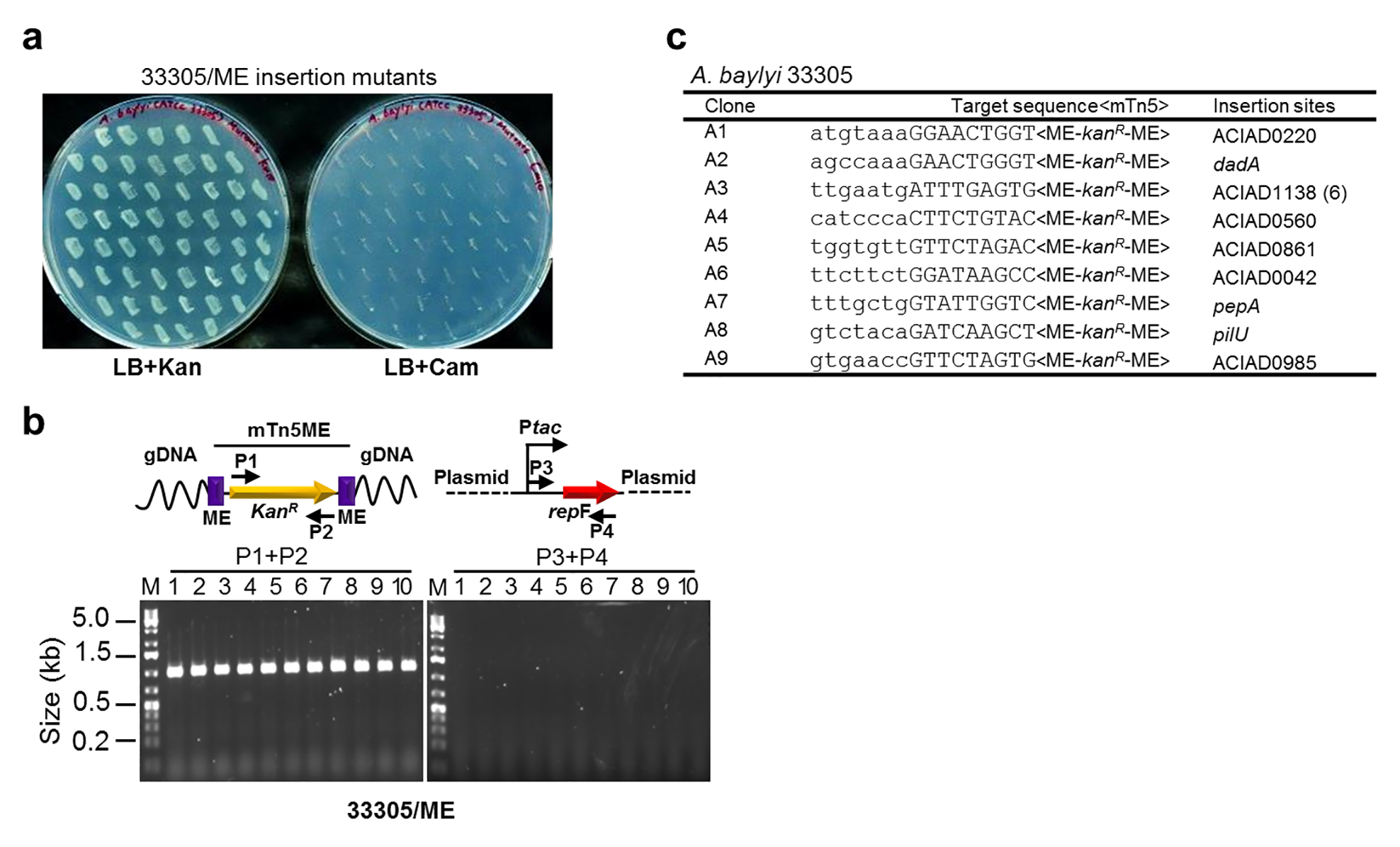

Supplement: Supplementary file 5 — Figure S5. Confirmation of mTn5ME transposition events in A. baylyi 33,305. (a) Colony restreaking. 100 random SucRKanR colonies of A. baylyi were restreaked on LB + Kan and LB + Cam plates. 100/100 were found to be KanRCamS and 50 restreaked colonies are shown here. (b) Colony PCR of ten restreaked clones in (a). All were found to be Tn5-positive and plasmid-negative. (c) Sequence analysis shows that 9/14 A. baylyi clones had different Tn5 insertion sites. (TIF 6938 kb) [file 12866_2018_1319_MOESM5_ESM.tif]

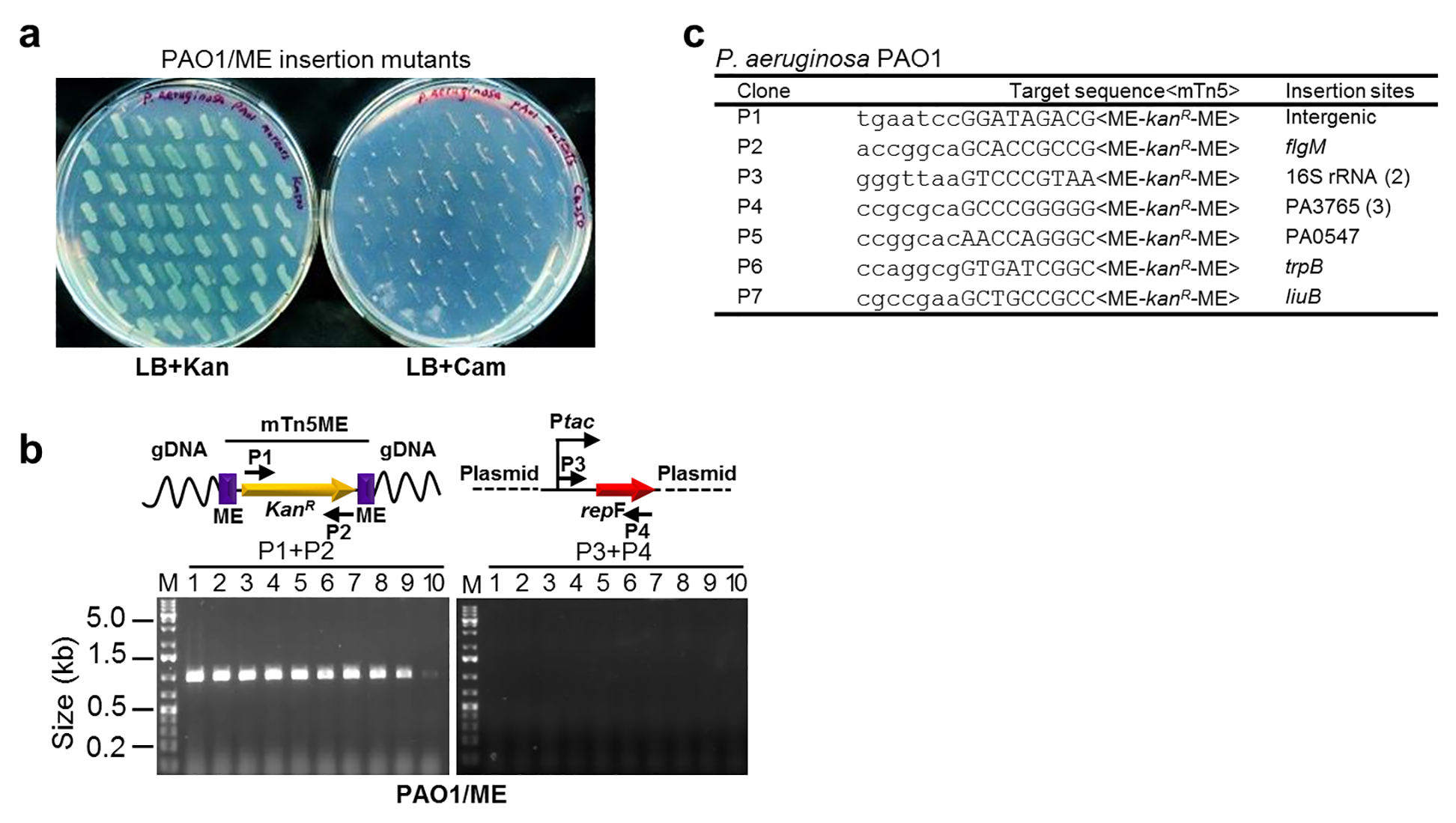

Supplement: Supplementary file 6 — Figure S6. Confirmation of mTn5ME transposition events in P. aeruginosa PAO1. (a) Colony restreaking. 100 random SucRKanR colonies of P. aeruginosa PAO1 were restreaked on LB + Kan and LB + Cam plates. 100/100 were found to be KanRCamS and 50 restreaked colonies are shown here. (b) Colony PCR of ten restreaked clones in (a). All were found to be Tn5-positive and plasmid-negative. (c) Sequence analysis shows that 7/10 P. aeruginosa clones had different Tn5 insertion sites. (TIF 6280 kb) [file 12866_2018_1319_MOESM6_ESM.tif]

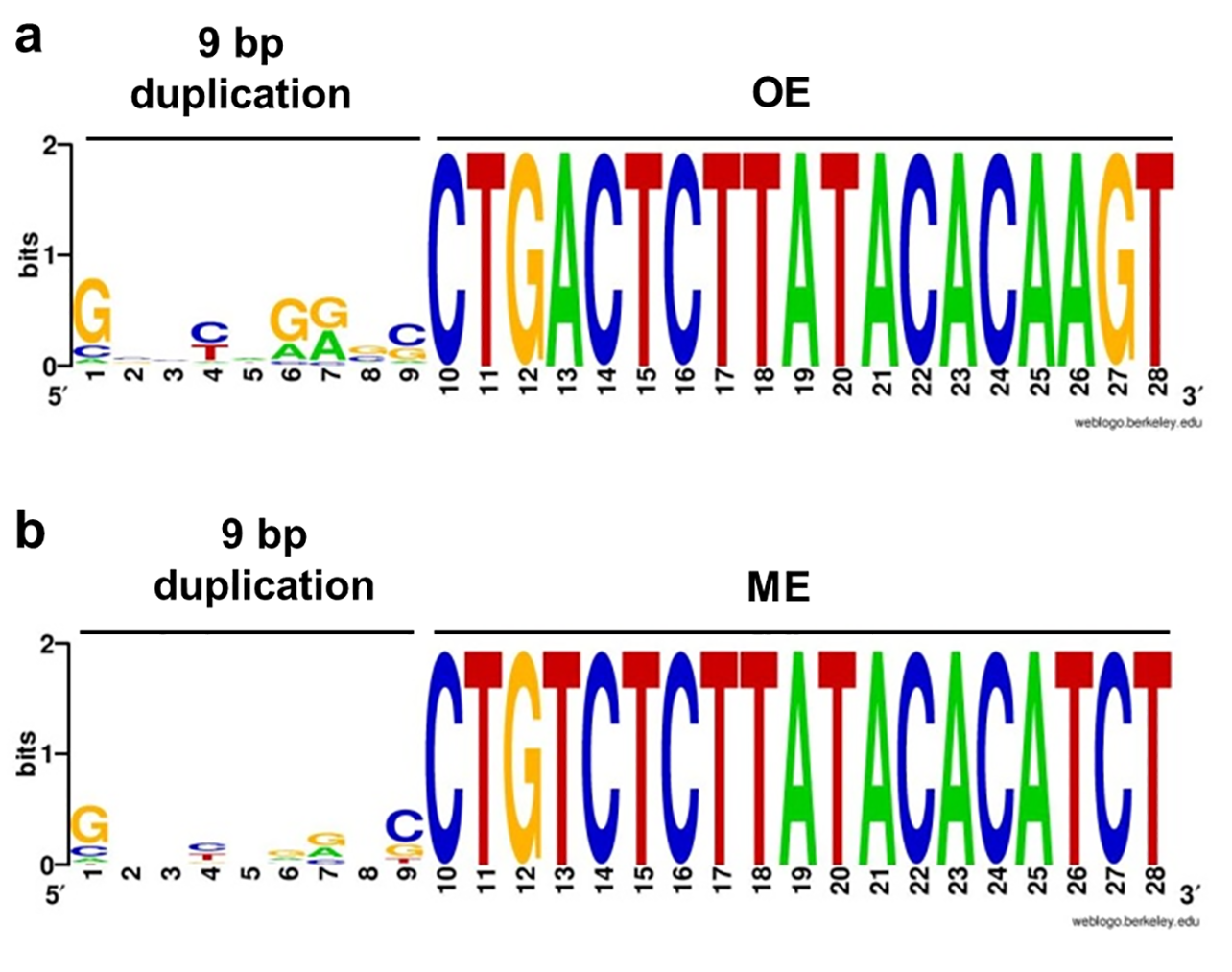

Supplement: Supplementary file 7 — Figure S7. Target site preferences of mTn5 and mTn5ME in P. aeruginosa. (a) Sequence logo of mTn5 insertion sites generated with WebLogo. 40 target sequences were analyzed. The 9 bp duplicated sequences adjacent to the OE are shown. There is a slight preference for certain nucleotides at several positions. (b) Sequence logo of mTn5ME insertion sites. 41 target sequences were analyzed. The 9 bp duplicated sequences adjacent to an ME are shown. It appears that mTn5ME has less nucleotide preference at the duplicated target sequence than mTn5 in Pseudomonas. (TIF 3834 kb) [file 12866_2018_1319_MOESM7_ESM.tif]

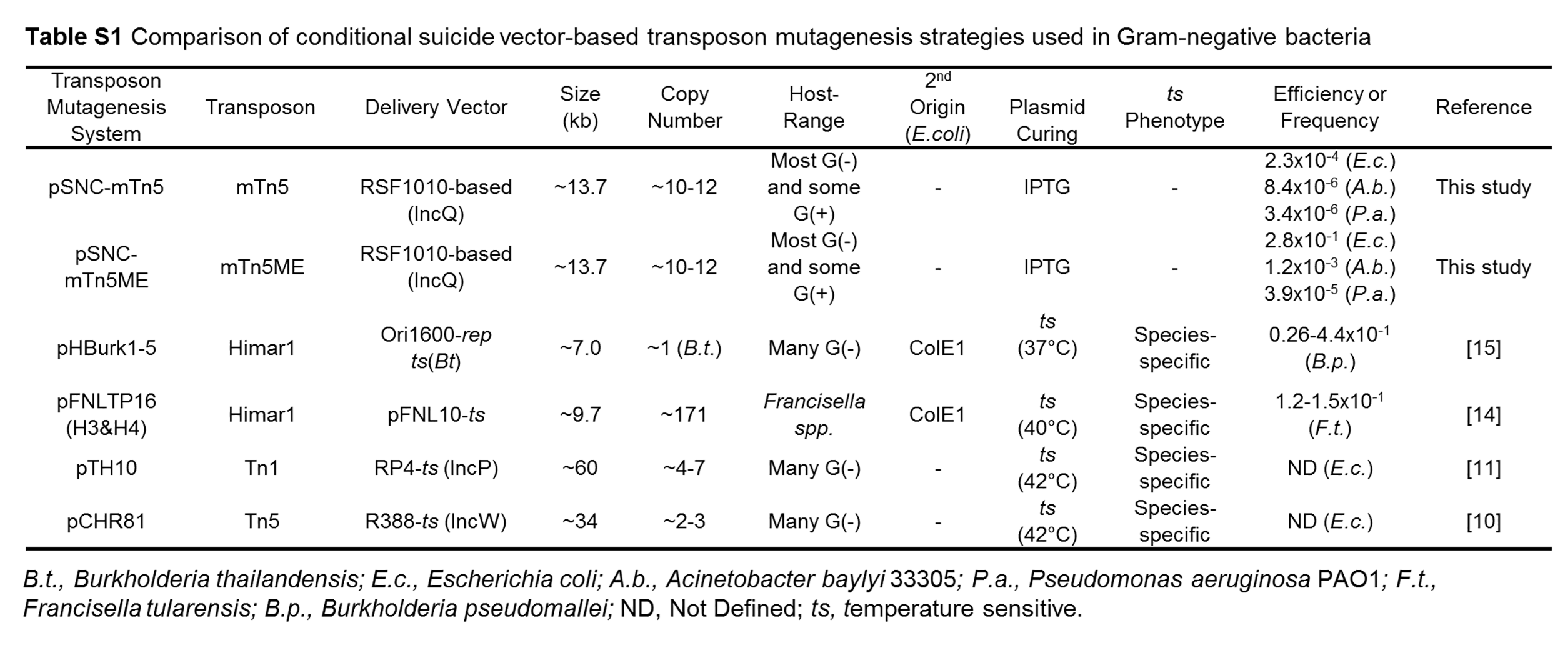

Supplement: Supplementary file 8 — Table S1. Comparison of conditional suicide vector-based transposon mutagenesis strategies used in Gram-negative bacteria. (TIF 7782 kb) [file 12866_2018_1319_MOESM8_ESM.tif]

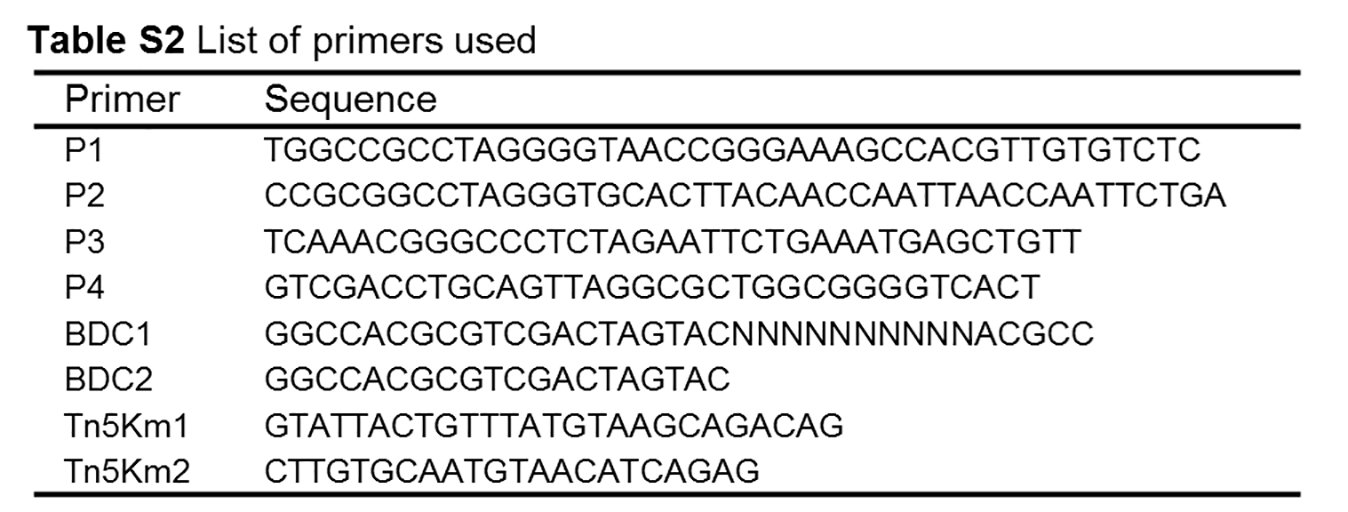

Supplement: Supplementary file 9 — Table S2. List of primers used. (TIF 2042 kb) [file 12866_2018_1319_MOESM9_ESM.tif]
